# Supplementary material for: An Exploratory Critical Review on TNF-α as a Potential Inflammatory Biomarker Responsive to Dietary Intervention with Bioactive Foods and Derived Products
Source: Foods. 2022 Aug 21;11(16):2524. doi: 10.3390/foods11162524 (PMC9407274; doi:10.3390/foods11162524)
Supplement: Supplementary file 1 [file foods-11-02524-s001.zip › foods-1864095-supplementary.pdf]

Table S1.- Reported differences in the circulatory levels of TNF-α between lean, overweight and obese individuals.

	Sample population		TNF-α levels				
Ref.	Country<br>Sex	Body weight phenotype + co-morbidities group,<br>Age[a] (y)	Type of sample<br>Method	Results per group[a] (*N*)[b]	CV%[c]	(Sig)<br>Effect<br>(% change *vs.* Lean)	General message conveyed by the article
[21]	Sweden<br>Women	Lean, 34.4 ± 1.2<br>Obese, 34.4 ± 1.2	Plasma,<br>ELISA	Protein levels (pg/mL)<br>Lean (*2*): 1.9 ± 0.4<br>Obese (*2*): 3.2 ± 2.8	 22%<br>88%	 (NS)<br>↑ (+68%)	Obesity correlates with increased plasma TNF-α levels. (*N* = only 2 values).
[22]	Spain<br>Women	Lean, 38.4 ± 2.3<br>Obese, 46.3 ± 1.3<br>Obese + T2DM, 51.6 ± 2.6<br>Very obese, 41.5 ± 2.2<br>Very obese + T2DM, 46.3 ± 1.8	Plasma,<br>ELISA	Protein levels (pg/mL)<br>Lean (*24*): 5.7 ± 9.8<br>Obese (*63*): 11.6 ± 19.0<br>Obese + T2DM (*19*): 15.3 ± 17.4<br>Very obese (*29*): 8.6 ± 12.4<br>Very obese + T2DM (*22*): 12.0 ± 15.0	>100%	 (NS)<br>↑ (+104%)<br>↑ (+168%)<br>↑ (+51%)<br>↑ (+111%)	Increased levels of TNF-α with BMI. Very high variability in the results.
[23]	Poland<br>Women	Lean, 29.4 ± 9.4<br>Overweight, 32.9 ± 11.3<br>Obese, 40.7 ± 11.0<br>Very obese, 40.9 ± 10.4	Serum,<br>ELISA	Protein levels (pg/mL)<br>Lean (*28*): 2.9 ± 2.2<br>Overweight (*24*): 6.5 ± 3.1<br>Obese (*81*): 6.8 ± 3.1<br>Very obese (*21*): 7.4 ± 2.6	 35%<br>46%<br>48%<br>76%	 (*p*<0.001)<br>↑ (+124%)<br>↑ (+135%)<br>↑ (+155%)	Obesity associates with increased serum TNF-α levels.
[24]	Israel<br>Mixed	Non-obese, 41.9 ± 7.6<br>Obese, 43.1 ± 12.3	Serum,<br>ELISA	Protein levels (pg/mL)<br>Non-obese (*9*), 0.3 ± 0.3<br>Obese (*41*), 1.0 ± 0.8	 100%<br>80%	 (*p*<0.001)<br>↑ (+233%)	Obesity associates with increased serum TNF-α levels.
[25]	Korea<br>Mixed	Non obese, 15.4 ± 0.5<br>Obese, 15.3 ± 0.5	Serum,<br>ELISA	Protein levels (pg/mL)<br>Non-obese (*32*), 5.9 ± 2.0<br>Obese (*39*), 18.2 ± 6.7	 33%<br>37%	 (*p*<0.001)<br>↑ (+208%)	Obesity associates with increased serum TNF-α levels.
[26]	USA<br>Women	Lean, 36.9 ± 11.8<br>Obese, 43.2 ± 8.6	Plasma,<br>ELISA	Protein levels (pg/mL)<br>Lean (*16*), 2.7 ± 0.9<br>Obese (*16*), 3.6 ± 1.8	 34%<br>50%	 (*p*<0.05)<br>↑ (+33%)	Obesity associates with increased serum TNF-α levels.
[27]	USA<br>Mixed	Lean, 42.2 ± 0.2<br>Obese, 45.2 ± 0.2	Plasma,<br>ELISA	Protein levels (pg/mL)<br>Lean (*84*), 3.8 ± 1.6<br>Obese (*84*), 4.3 ± 1.6	 42%<br>36%	 (*p*<0.05)<br>↑ (+13%)	Obesity associates with increased serum TNF-α levels.
[28]	USA<br>Mixed	Lean, 11.9 ± 4.0<br>Obese, 11.6 ± 3.4	Serum,<br>ELISA	Protein levels (pg/mL)<br>Lean (*6*), 3.1 ± 1.8<br>Obese (*21*), 4.2 ± 2.0	 58%<br>48%	 (NS)<br>↑ (+35%)	Serum TNF-α levels is higher in obese but the differences didn't reach significance.

[29]	Netherlands<br>Mixed	Lean, 38.3 ± 2.1<br>Obese, 37.4 ± 1.2	Plasma,<br>ELISA	Protein levels (ng/L = pg/mL)<br>Lean (*13*), 3.4 ± 0.4<br>Obese (*15*), 4.5 ± 0.5	 12%<br>11%	 (NS)<br>↑ (+32%)	Serum TNF-α levels is higher in obese but the differences didn't reach significance.
[30]	Poland<br>Women	Lean, 32.6 ± 8.6<br>Obese, 30.1 ± 5.5	Serum,<br>ELISA	Protein levels (pg/mL)<br>Lean (*15*), 4.5 ± 2.3<br>Obese (*26*), 8.8 ± 7.0	 50%<br>80%	 (p<0.05)<br>↑ (+96%)	Obesity associates with increased serum TNF-α levels.
[31]	USA<br>Women	Lean, 31.9 ± 5.8<br>Obese, 35.7 ± 4.1<br>Obese + PCOS, 29.6 ± 5.8	Plasma,<br>ELISA	Protein levels (pg/mL)<br>Lean (*15*), 3.1 ± 0.7<br>Obese (*17*), 3.8 ± 0.8<br>Obese + PCOS (*42*), 4.1 ± 1.9	 22%<br>21%<br>47%	 (NS)<br>↑ (+23%)<br>↑ (+32%)	Plasma TNF-α levels are higher in obese but the differences didn't reach significance.
[32]	USA<br>Mixed	Lean, 35.0<br>Obese, 46.0	Serum,<br>ELISA	Protein levels (pg/mL)<br>Lean (*10*), 82.3 ± 89<br>Obese (*9*), 0.8 ± 7.2	 >100%<br>>100%	 (p=0.015)<br>↓ (-99%)	This study indicates opposite results to all the other ones.<br>Very high variability in the data, especially in the obese participants.
[33]	New Zealand<br>Women	Lean, 53 ± 10<br>Obese, 54 ± 9	Plasma<br>ELISA	Protein levels (ng/L = pg/mL)<br>Lean (*14*), 0.8 ± 0.4<br>Obese (15), 1.6 ± 1.0	 50%<br>62.5%	 (p=0.046)<br>↑ (+100%)	Obese women had higher levels of TNF-α.
[34]	USA<br>Mixed	Lean, 40.0 ± 14 .9<br>Obese + T2DM, 50 ± 10.8	Plasma,<br>ELISA	Protein levels (pg/mL)<br>Lean (*14*), 2.8 ± 0.5<br>Obese + T2DM (*13*), 4.3 ± 2.7	 17%<br>64%	 (p<0.05)<br>↑ (+53%)	Obesity associates with increased serum TNF-α levels.
[35]	Czech Republic,<br>Women	Lean, 37.7 ± 2.8<br>Obese, 39.4 ± 9.5	Plasma,<br>ELISA	Protein levels (pg/mL)<br>Lean (*12*), 0.6 ± 0.3<br>Obese (*20*), 0.7 ± 0.2	 50%<br>29%	 (p<0.05)<br>↑ (+16%)	Obesity associates with increased serum TNF-α levels. Data estimated from figures.
[36]	Canada<br>Men	Lean, 42.0 ± 10.7<br>Obese, 46.2 ± 7.1<br>Very obese, 43.0 ± 6.9	Plasma<br>ELISA	Protein levels (pg/mL)<br>Lean (*59*), 1.0 ± 1.1<br>Obese, (*101*), 1.1 ± 1.1<br>Very obese (*57*), 1.3 ± 1.1	 >100%<br>92%<br>90%	 (p<0.05)<br>↑ (+19%)<br>↑ (+30%)	TNF-α levels increased with BMI.
[37]	USA<br>NR	Lean, 43 ± 14.4<br>Overweight, 49 ± 12.6<br>Obese, 45 ± 20.8<br>Very obese, 42 ± 12.7<br>Morbidly obese, 41 ± 19.3	Plasma,<br>ELISA	Protein levels (pg/mL)<br>Lean (*13*), 39.0 ± 50.5<br>Overweight (*10*), NR<br>Obese (*12*), 294.0 ± 142.0<br>Very obese (*18*), 478.0 ± 233.3<br>Morbidly obese (*15*), 713.0 ± 240.1	 >100%<br>ND<br>48%<br>49%<br>34%	 (p<0.001)<br>ND<br>↑ (+653%)<br>↑ (+1125%)<br>↑ (+1728%)	TNF-α positively correlates with BMI.
[38]	Poland<br>Women	Lean, 36.4 ± 8.0<br>Overweight, 33.3 ± 12.0	Plasma,<br>ELISA	Protein levels (pg/mL)<br>Lean (*14*), 3.1 ± 3.0	 97%	 (p<0.001)	TNF-α positively correlates with BMI.

		Obese, 43.8 ± 10.8		Overweight (*12*), 6.3 ± 3.0<br>Obese (*57*), 6.9 ± 2.9	48%<br>42%	↑ (+103%)<br>↑ (+123%)	
[39]	Poland<br>Women	Lean, 38.0 ± 8.0<br>Obese, 52.0 ± 9.0<br>Obese + MetS, 53.0 ± 9.0	Plasma<br>ELISA	Protein levels (pg/mL)<br>Lean (*14*), 5.6 ± 2.0<br>Obese (*21*), 6.4 ± 5.5<br>Obese (*71*) + MetS, 6.3 ± 1.9	 38%<br>86%<br>30%	 (NS)<br>↑ (+14%)<br>↑ (+13%)	Obesity and MetS do not significantly associate with an increase in serum TNF-α levels.
[40]	Egypt<br>Mixed	Men<br>Lean, 32.4 ± 2.89<br>Class I obese, 33.1 ± 3.13<br>Class II obese, 32.5 ± 3.14<br>Class III obese, 32.9 ± 3.06<br>Women<br>Lean, 31.6 ± 3.15<br>Class I obese, 31.9 ± 3.0<br>Class II obese, 32.3 ± 3.4<br>Class III obese, 32.1 ± 3.1	Serum,<br>ELISA	Protein levels (pg/mL)<br>Men<br>Lean (*15*), 3.0 ± 0.7<br>Class I obese (*15*), 6.7 ± 1.5<br>Class II obese (15), 7.3 ± 1.9<br>Class III obese (*15*), 9.3 ± 2.0<br>Women<br>Lean (*15*), 2.8 ± 0.7<br>Class I obese (*15*), 4.8 ± 1.3<br>Class II obese (*15*), 5.1 ± 2.0<br>Class III obese (*15*), 7.3 ± 2.1	 <br>23%<br>22%<br>26%<br>21%<br><br>15%<br>27%<br>39%<br>29%	 <br>($p<0.05$)<br>↑ (+123%)<br>↑ (+143%)<br>↑ (+210%)<br><br>($p<0.05$)<br>↑ (+71%)<br>↑ (+82%)<br>↑ (+161%)	TNF-α positively correlates with BMI. Slightly higher TNF-α levels were detected in men than in women.
[41]	Brazil<br>Mixed	T2DM, 56.0 (12.0)<br>Non diabetes, 53.0 (18.0)<br>Lean<br>Lean + T2DM<br>Overweight<br>Overweight + T2DM<br>Obese<br>Obese + T2DM	Plasma<br>Cytometry Bed Array	Protein levels (pg/mL)<br>T2DM (*102*), Non diabetes (*62*)<br>Lean: 0.090 (0.075)<br>Lean + T2DM: 0.086 (0.048)<br>Overweight: 0.086 (0.068)<br>Overweight + T2DM: 0.092 (0.048)<br>Obese: 0.112 (0.058)<br>Obese + T2DM: 0.092 (0.042)	ND	 <br>(NS)<br>↓ (-4%)<br>↓ (-4%)<br>↑ (+2%)<br>↑ (+24%)<br>↑ (+2%)	Results converted from fg/mL to pg/mL. Very low values compared to other reports.
[42]	Pakistan<br>Mixed	Lean, 46.5 ± 8.2<br>Overweight + MetS, 47.08 ± 8.0	Serum<br>ELISA	Protein levels (pg/mL)<br>Lean (*200*): 9.5 (6.1 – 18.0)<br>Overweight + MetS: 20.5 (10.0 – 62.0)	ND	 ($p<0.0001$)<br>↑ (+115%)	Increased levels of TNF-α with increased BMI.
[43]	Australia<br>Mixed	Lean, 33.0 ± 3.2<br>Overweight, 44.9 ± 4.2<br>Obese, 43.3 ± 4.5	Serum<br>ELISA	Protein levels (pg/mL)<br>Lean (*12*): 3.6 ± 1.2<br>Overweight (*9*): 3.1 ± 3.9<br>Obese (*8*): 4.2 ± 2.3	 33%<br>>100%<br>55%	 (NS)<br>↓ (-14%)<br>↑ (17%)	No significant differences between phenotypes. Results estimated from figure.
[44]	Spain<br>Mixed	Lean, 65.6 ± 0.7<br>Overweight, 64.9 ± 0.7<br>Obese, 64.0 ± 0.6	Plasma<br>ELISA	Protein levels (pg/mL)<br>Lean (*50*): 25.0 ± 11.0<br>Overweight (*50*): 30.0 ± 11.0<br>Obese (*50*): 34.0 ± 14.0	 41%<br>37%<br>44%	 (NS)<br>↑ (+20%)<br>($p<0.001$)<br>↑ (+36%)	TNF-α increased with BMI. Results estimated from figure.

[45]	Poland<br>Men	Lean, 21.4 ± 1.2<br>Class I obese, 31.1 ± 3.8<br>Class II obese, 28.4 ± 3.6	Serum,<br>ELISA	Protein levels (pg/mL)<br>Lean (*9*): 3.5 ± 1.7<br>Obese (I) (*9*): 3.5 ± 0.9<br>Obese (II): 5.2 ± 1.3	 49%<br>26%<br>25%	 (NS)<br>NC<br>↑ (+49%)	Data estimated from bar figure. No significant differences between the groups.

Legend: [a]: Most values presented as the mean ± standard deviation (SD), some data indicated as Median (Inter Quartile Range, IQR); [b]: (*N*): sample size per group; [c]: CV%: Coefficient of variation in percentage estimated for each subgroup ((CV%) = (Standard Deviation/Mean) × 100); BMI: Body mass index; MetS: Metabolic syndrome; NC: No change; ND: Not determined; NR: Not reported; NS: Not significant; PCOS: Polycystic Ovary Syndrome; T2DMM: Type 2 diabetes mellitus; TNF-α: Tumour necrosis factor alpha.

Table S2.- Reported changes in the circulatory levels of human TNF-*α* following dietary intervention with PUFAs-containing foods and products.

	Intervention	Sample population		TNF-*α* levels				
Ref.	Tested product/main compounds<br>Study design	Country<br>Sex	Body weight phenotype and comorbidities groups,<br>Age[a] (y)	Type of Sample Method	Results per group[a] (*N*)[b]	CV%[c]<br>per group	Sig<br>Effect<br>(%A *vs* B per group[d];<br>T – C[e] pg/mL)	General messages conveyed by the article
PUFAs (ω-3 PUFAs: EPA, DHA in capsules, oils)								
[63]	T: ω-3 PUFAs (1110 mg/d: 600 mg/d EPA and 510 mg/d DHA)<br>C: PLA (sunflower oil)<br>RCT (crossover), 42 d (14 d washout phase)	The Netherlands<br>Men	Obese healthy<br>59.0 ± 9.0	Plasma<br>ELISA	Protein levels (pg/mL)<br><br>T: ω-3 PUFAs (*11*)<br>0 h: 1.09 ± 0.54;<br>2 h: 1.39 ± 0.78;<br>4 h: 1.10 ± 0.65<br><br>C: PLA (*11*)<br>0 h: 1.11 ± 0.64;<br>2 h: 1.31 ± 0.57;<br>4 h: 1.37 ± 0.68<br><br>T - C	 <br><br>49%<br>56%<br>59%<br><br><br>57%<br>43%<br>49%	 <br><br>(NS)<br>+28%<br>+1%<br><br><br>(NS)<br>+18%<br>+23%<br><br>-0.02 pg/mL (NS)	No significant differences in the levels of TNF-*α* between T and C at any time point.<br>No effects on body weight.

[64]	T: ω-3 PUFAs (1200 mg/d: 930 mg EPA, 290 mg DHA, 100 mg GLA, 18 mg Vitamin E/d)<br>C: PLA (medium-chain triglycerides)<br>RCT (crossover), 90 d (21 d washout phase)	USA<br>Mixed	Obese<br>15.7 ± 1.0	Serum<br>ELISA	Protein levels (pg/mL)<br>T: ω-3 PUFAs (*25*)<br>B: NR; A: 5.20 ± 1.0<br>C: PLA (*25*)<br>B: NR; A: 5.70 ± 1.50<br>T – C	ND; 19%<br>ND; 26%	ND<br>ND<br>-0.50 pg/mL (*p*=0.008, based only on values after (A) intervention)	Significant but small reduction of the levels of TNF-*α* with T.<br>No effect on body weight
[65]	T: Fish oil (900 mg/d: 360 mg DHA and 540 mg EPA)<br>C: PLA (corn starch)<br>RCT (parallel), 30 d	Mexico<br>Mixed	Overweight, IR<br>T: 13.6 ± 1.9<br>C: 12.9 ± 2.0	Serum<br>ELISA	Protein levels (pg/mL)<br>T: Fish oil (*49*)<br>B: 11.35 ± 4.23; A: NR<br>C: PLA (*27*)<br>B: 10.13 ± 4.98; A: NR<br>T – C	37%; ND<br>49%; ND	ND<br>ND<br>NR (*p*=0.052)	Significant reduction of the levels of TNF-*α* but values not clearly indicated. No information on data after intervention.<br>Effect on body weight only in some children. Some numeric data are missing.
[66]	T: Fish oil (4000 mg/d: 1860 mg EPA and 1500 mg DHA)	USA<br>Mixed	Obese, IR nondiabetic<br>T: 48.8 ± 2.3	Plasma<br>ELISA	Protein levels (pg/mL)			No significant effects on the levels of TNF-*α*.

	C: PLA (corn oil)<br>RCT (parallel), 84 d		C: 53.3 ± 2.2		T: Fish oil (*19*)<br>B: 17.6 ± 2.9; A: 22.3 ± 3.4<br>C: PLA (*14*)<br>B: 25.8 ± 4.2; A: 19.6 ±2.6<br>T – C	16%; 15%<br>16%; 13%	(NS)<br>+26.7%<br>(NS)<br>-24.03%<br>+10.9 pg/mL (NS)	No effects on body weight.
[67]	T: DHA (500 mg/d during the first 60 d and 250 mg/d till the end of the study) + VLCK diet<br>C: PLA + VLCK diet<br>RCT (parallel), 180 d	Spain<br>Mixed	Obese<br>T: 47.4 ± 9.1<br>C: 44.3 ± 11.7	Serum<br>ELISA	Protein levels (pg/mL)<br>T: VLCK diet (*14*)<br>B: 5.0 ± 2.5; A: 2.0 ± 0.3<br>C: VLCK diet (*15*)<br>B: 4.7 ± 2.3; A: 2.0 ± 0.4<br>T – C	50%; 15%<br>49%; 20%	(NS)<br>-60%<br>(NS)<br>-57%<br>-0.3 pg/mL (NS)	No significant effects on the levels of TNF-α. Poor indication of TNF-α units.<br>No significant differences in body weight loss between groups.
PUFAs-containing foods								
[68]	T: chia seeds (50 g/d)<br>C: PLA<br>RCT (parallel), 84 d	USA<br>Mixed	Overweight/obese<br>20 - 70	Plasma<br>ELISA	Protein levels (pg/mL)<br>*Men*<br>T: Chia seeds (*14*)<br>B: 1.63 ± 1.12; A: 1.52 ± 1.01	69%; 66%	(NS)<br>-7%	No effects on the levels of TNF-α.<br>No effect on body weight.

					C: PLA (*14*)<br>B: 1.59 ± 1.12; A: 1.39 ± 0.52<br><br>T - C<br><br>*Women*<br>T: Chia seeds (*25*)<br>B: 1.48 ± 1.25; A: 1.6 ± 1.25<br><br>C: PLA (*23*)<br>B: 1.4 ± 0.96; A: 1.41 ± 0.76<br><br>T - C	 70%; 37%<br><br><br><br><br><br>85%; 78%<br><br><br>69%; 54%	(NS)<br>-12%<br><br>+0.11 pg/mL (NS)<br><br><br>(NS)<br>+8%<br><br>(NS)<br>0%<br><br>+0.11 pg/mL (NS)	
[69]	T: Flaxseeds (40 g/d)<br>C: PLA<br>RCT (crossover), 84 d (28 d, washout phase)	USA<br>NR	Obese, glucose intolerant<br>54.7 ± 6.6.	Plasma<br>ELISA	Protein levels (pg/mL)<br><br>T: Flaxseeds (*7*)<br>B: 0.9 ± 0.4; A: 0.8 ± 0.5<br><br>C: PLA (*7*)<br>B: 0.6 ± 0.5; A: 0.7 ± 0.6<br><br>T - C	 <br><br>44% 62%<br><br><br>83% 86%	 <br>(NS)<br>-11%<br><br>(NS)<br>+13%<br><br>-0.2 pg/mL (NS)	No significant effect on the levels of TNF-α.<br>No effects on body weight.

[70]	T: *Nigella sativa* oil<br>(3 g/d) + LCD,<br>C: PLA + LCD<br>RCT (parallel), 56 d	Iran<br>Women	Obese,<br>T: 41 ± 11.8<br>C: 39.5 ± 9.8	Serum<br>ELISA	Protein levels (pg/mL)<br><br>T: *Nigella sativa* oil (*45*)<br>B: 24.0 ± 17.3; A: 14.2 ± 10.9<br><br>C: PLA (*45*)<br>B: 21.7 ± 15.2; A: 18.2 ± 13.5<br><br>T - C	 <br><br>72%, 76%<br><br><br>70%, 74%	 <br>(*p*<0.05)<br>-41%<br><br>(NS)<br>-16%<br><br>-6.3 pg/mL(*p*=0.03)	Significant reduction of the levels of TNF-*α* with T.<br>No effects on body weight.
[71]	T: Baru almonds (20 g/d rich in MUFAs 51% and PUFAs 31%);<br>C: PLA<br>RCT (parallel), 56 d	Brazil<br>Women	Overweight/obese<br>40.0 ± 11.0	Plasma<br>Milliplex	Protein levels (pg/mL)<br><br>T: Almonds (*24*)<br>B: 2.1 ± 0.1; A: 2.2 ± 0.1<br><br>C: PLA (*22*)<br>B: 2.6 ± 0.3; A: 2.0 ± 0.1<br><br>T – C	 <br><br>5%; 5%<br><br><br>16%; 5%	 <br>(NS)<br>+5%<br><br>(NS)<br>-23%<br><br>+0.7 pg/mL (NS)	No significant effects on the levels of TNF-*α*.<br>No effects on body weight.

Legend: [a]: Values are presented as the mean ± SD; [b]:(*N*): Sample size per group; [c]: CV%: Coefficient of variation in percentage estimated for each analyzed subgroup ((CV%) = (Standard Deviation/Mean) × 100); B: Before intervention; A: After intervention; [d]:Difference in % before and after intervention; T: Treatment; C: Control; T – C[e]: effect size calculated as the difference between the T and the C group (pg/mL); DHA: Docosahexaenoic acid; EPA: Eicosapentaenoic acid; GLA: Gamma-linolenic acid; IR: Insulin resistance; LCD: Low calorie diet; MUFAs: Monounsaturated fatty acids; ND: Not determined; NR: Not reported; NS: Not significant; PLA: Placebo; PUFAs: Polyunsaturated fatty acids; RCT: Randomized clinical trial; TNF-*α*: tumor necrosis factor alpha; VLCK: Very low-calorie ketogenic diet.

Table S3.- Reported changes in the circulatory levels of human TNF-α following dietary intervention with VitD supplements.

	Intervention	Sample population		TNF-α levels				
Ref.	Tested product/main compounds<br>Study design	Country<br>Sex	Body weight phenotype and comorbidities groups<br>Age[a] (y)	Type of Sample<br>Method	Results per group[a] (*N*)[b]	CV%[c] per group	(Sig<br>Effect<br>(% A *vs* B per group[d];<br>T – C[e] pg/mL)	General messages conveyed by the article
[90]	T: Oily VitD preparation (3332 IU/d) + weight reduction program<br>C: PLA + weight reduction program<br>RCT (parallel), 365 d	Germany<br>Mixed	Overweight<br>T: 47.4 ± 10.0<br>C: 48.8 ± 10.0	Serum<br>Automated immunoassay	Protein levels (pg/mL)<br>T: Vitamin D + weight reduction program (*82*)<br>B: 7.84 ± 3.15; A: 7.04 ± 2.25<br>C: PLA + weight reduction program (*83*)<br>B: 8.12 ± 3.43; A: 7.90 ± 2.80<br>T - C	40%; 32%<br>42%; 35%	$p<0.05$<br>-10%<br>(NS)<br>-2.7%<br>-0.58 pg/mL ($p<0.05$)	Significant but rather small reduction of the levels of TNF-α with T.<br>No effect on body weight.
[91]	T: VitD (4000 IU/d) + exercise training program<br>C: PLA + exercise training program;<br>RCR (parallel), 84 d	USA<br>Mixed	Overweight/obese<br>T: 26.2 ± 5.1<br>C: 26.0 ± 4.5	Serum<br>ELISA	Protein levels (pg/mL)<br>T: Vitamin D + exercise (*10*)<br>B: 2.3 ± 1.1; A: 2.1 ± 1.2	48%; 57%	NS<br>-8.7%	No significant effect on the levels of TNF-α.<br>No effect on body weight.

					C: PLA + exercise (*13*)		NS	
					B: 1.9 ± 1.3; A: 1.9 ± 1.2	68%; 63%	-0%	
					T - C		-0.2 pg/mL (NS)	
				Supernatant of whole blood<br>ELISA	Protein levels (pg/mL)			
					T: Vitamin D + exercise (*10*)		*p*<0.05	
					B: 1.5 ± 0.6; A: 0.6 ± 0.5	40%; 83%	-60%	
					C: PLA + exercise (*13*)		*p*<0.05	
					B: 1.6 ± 0.9; A: 0.6 ± 0.5	56%; 83%	-62%	
					T - C		+0.1 pg/mL (NS)	
[92]	T: VitD (4000 IU/d)<br>C: PLA;<br>RCT (parallel), 183 d	USA<br>Mixed	Obese and IR<br>T: 14.6 ± 2.3<br>C: 13.9 ± 2.4	Serum<br>ELISA	Protein levels (pg/mL)			No significant reduction in TNF-α levels.<br>No effect on body weight.
					T: Vitamin D (*14*)		NS	
					B: 1.85 ± 0.15; A: 1.6 ± 0.15	8%; 9%	-13%	
					C: PLA (*15*)		NS	
					B: 1.7 ± 0.15; A: 1.4 ± 0.1	9%; 7%	-17%	
					T - C		+ 0.05 pg/mL (NS)	

[93]	T: Alphacalcidol (1 μg/d 1-α hydroxy-Vitamin D3)<br>C: PLA<br>RCT (parallel), 56 d	Iran<br>Mixed	Obese<br>T: 39 ± 8.9<br>C: 41 ± 14.5	Serum<br>ELISA	Protein levels (pg/mL)<br>T: Alphacalcidol (*40*)<br>B: 9.83 ± 25.42; A: NR<br>C: PLA (*54*)<br>B: 6.65 ± 14.10; A: NR<br>T – C	>100%; ND<br>>100%; ND	NS<br>-20%<br>NS<br>+25%<br>-3.5 pg/mL (NS)	No significant effect on the levels of TNF-α. Results estimated from Figures.<br>No effect on body weight.
[94]	T: VitD (initial bolus dose of 100,000 IU/d followed by 4,000 IU/d)<br>C: PLA<br>RCT (parallel), 112 d	Australia<br>Mixed	Overweight/obese<br>T: 30.5 (25, 35)<br>C: 29.5 (25, 41)	Serum<br>ELISA	Protein levels (pg/mL)<br>T: Vitamin D (*28*)<br>B: 29.6 (14.6, 55.0);<br>A: 24.3 (11.1, 45.6)<br>C: PLA (*26*)<br>B: 27.6 (16.9, 72.9);<br>A: 21.5 (12.4, 47.4)<br>T – C	ND<br>ND	NS<br>-18%<br>NS<br>-22%<br>+ 0.8 pg/mL (NS)	No significant effect on the levels of TNF-α.<br>No effect on body weight.

Legend: [a]: Values are generally presented as the mean ± standard deviation (SD), some data indicated as Median (Inter Quartile Range, IQR); [b]:(*N*): Sample size per group; [c]: CV%: Coefficient of variation in percentage estimated for each analyzed subgroup ((CV%) = (Standard Deviation/Mean) × 100); B: Before intervention; A: After intervention; [d]:Difference in % before and after intervention; T: Treatment; C: Control; T – C[e]: effect size calculated as the difference between the T and the C group (pg/mL); IR: Insulin resistance; ND: Not determined; NR: Not reported; NS: Not significant; PLA: Placebo; RCT: Randomized clinical trial; TNF-α: tumor necrosis factor alpha; VitD: Vitamin D.

Table S4.- Reported changes in the circulatory levels of human TNF-α following dietary intervention with products containing mixed bioactive (micro)nutrients.

	Intervention	Sample population		TNF-α levels				
Ref.	Tested product/main compounds<br>Study design	Country<br>Sex	Body weight phenotype and comorbidities groups<br>Age[a] (y)	Type of Sample<br>Method	Results per group[a] (*N*)[b]	CV%[c]	(Sig)<br>Effect<br>(% A vs B[d] per group<br>T – C[e] pg/mL)	General message conveyed by the article
[95]	T: NuFit active blend (3 times/d, 2.25g leucine and 30 mg vitamin B6),<br>C: PLA<br>RCT (parallel), 28 d	USA<br>Mixed	Overweight/obese<br>T: 33 ± 5.1<br>C: 25 ± 5.8	Plasma<br>ELISA	Protein levels (pg/mL)<br><br>T: NuFit (*10*)<br>B: 408 ± 22; A: 334 ± 38<br><br>C: PLA (*10*)<br>B: 411 ± 40; A: 393 ± 29<br><br>T - C	 <br><br>5%; 11%<br><br><br>10%; 7%	 <br>(*p*<0.01)<br>-18%<br><br>(NS)<br>-4%<br><br>-56 pg/mL (NR)	Reduction in TNF-α levels.<br>No effects on body weight.
[96]	T: L-arginine (9 g/d)<br>C: PLA<br>RCT (parallel), 90 d	Poland<br>Mixed	Obese<br>T: 43.8 ± 8.2<br>C: 41.0 ± 8.8	Serum<br>ELISA	Protein levels (pg/mL)<br><br>T: L-arginine (*30*)<br>B: 4.6 ± 2.0; A: 4.3 ± 1.8<br><br>C: PLA (*30*)<br>B: 4.6 ± 1.8; A: 4.5 ± 1.5<br><br>T - C	 <br><br>43%; 41%<br><br><br>39%; 33%	 <br>(NS)<br>-7%<br><br>(NS)<br>-2%<br><br>-0.2 pg/mL (NS)	Non-significant tendency to decrease of TNF-α levels.<br>No effects on body weight.
[97]	T: Black soy peptide (4.5 g/d)<br>C: PLA<br>RCT (parallel), 84 d	Korea<br>Mixed	Overweight/obese<br>T: 39 ± 10.3<br>C: 36.2 ± 8.1	Serum<br>ELISA	Protein levels (pg/mL)<br><br>T: Black soy peptide (*35*)<br>B: 4.84 ± 0.67; A: 3.97 ± 0.65<br><br>C: PLA (*29*)<br>B: 5.97 ± 0.97; A: 4.49 ± 0.78<br><br>T - C	 <br><br>14%; 16%<br><br><br>16%; 17%	 <br>(*p*= 0.017)<br>-18%<br><br>(NS)<br>-25%<br><br>+0.61 pg/mL (NR)	Reduction of TNF-α levels in the T group.<br>Significant reduction of body weight.
[98]	T: Zinc (30 mg/d)<br>C: PLA<br>RCT (parallel), 56 d	Korea<br>Women	Obese<br>20.8 ± 2.2	Serum<br>ELISA	Protein levels (pg/mL)<br><br>T: Zinc (*20*)<br>B: 16.1 ± 7.1; A: 13.9 ± 4.9<br><br>C: PLA (*20*)<br>B: 13.0 ± 5.0; A: 14.7 ± 4.7	 <br><br>44%; 35%<br><br><br>38%; 32%	 <br>(NS)<br>-14%<br><br>(NS)<br>+13%	No reduction in TNF-α levels.<br>No effects on body weight.

					T - C		-3.9 pg/mL (NS)	
[99]	T: Yeast β–Glucan 1 capsule/d for the first 14 d followed by 2 capsule/d for the other 28 d (477 mg/capsule)<br>C: PLA<br>RCT, 42 d	Thailand<br>Mixed	Overweight/obese<br>41.3 ± 10.7	Serum<br>ELISA	Protein levels (pg/mL)<br><br>T: Yeast β–Glucan 1 (*22*)<br>B: 10.5 ± 5; A: 10.2 ± 4<br><br>C: PLA (*22*)<br>B: 10.2 ± 3; A: 10.5 ± 1.5<br><br>T - C	 <br><br>47%; 40%<br><br><br>29%; 14%	 <br>(NS)<br>-3%<br><br>(NS)<br>+3%<br><br>-0.6 pg/mL (NS)	Significant reduction of TNF-*α* levels (only after 14 d).<br>Reduction of waist circumference.
[100]	T: Hydrolyzed cod proteins (4 g/d)<br>C: PLA<br>RCT (parallel), 56 d	Norway<br>Mixed	Obese, MetS<br>T: 52.8 ± 6.2<br>C: 53.4 ± 6.8	Serum<br>ELISA	Protein levels (pg/mL)<br><br>T: Cod proteins (*15*)<br>B: 0.57 (0.22, 0.93)<br>A: 0.93 (0.22, 0.93)<br><br>C: PLA (*15*)<br>B: 0.22 (0.11, 0.93)<br>A: 0.57 (0.22, 0.93)<br><br>T - C	 <br><br>ND<br>ND<br><br><br>ND<br>ND	 <br>(NS)<br>+63%<br><br><br>(NS)<br>+159%<br><br><br>+0.01 pg/mL (NS)	No effect on the levels of TNF-*α*.<br>No effects on body weight.

Legend: [a]: Values are generally presented as the mean ± standard deviation (SD), some data indicated as Median (Inter Quartile Range, IQR); [b]:(*N*): Sample size per group; [c]: CV%: Coefficient of variation in percentage estimated for each analyzed subgroup ((CV%) = (Standard Deviation/Mean) × 100); B: Before intervention; A: After intervention; [d]:Difference in % before and after intervention; T: Treatment; C: Control; T – C[e]: effect size calculated as the difference between the T and the C group (pg/mL); MetS: Metabolic syndrome; ND: Not determined; NR: Not reported; NS: Not significant; PLA: Placebo; RCT: Randomized clinical trial; TNF-*α*: tumour necrosis factor alpha.

Table S5. Reported changes in the circulatory levels of human TNF-α following dietary intervention with different products containing polyphenols and other phytochemicals.

	Intervention	Sample population		TNF-α levels				
Ref.	Tested product/main compounds<br>Study design	Country<br>Sex	Body weight phenotype and comorbidities groups<br>Age[a] (y)	Type of Sample<br>Method	Results per group[a] (*N*)[b]	CV%[c]	(Sig)<br>Effect<br>(% A *vs* B[d] per group<br>T – C[e] pg/mL)	General message conveyed by the article
Polyphenol-containing products								
[114]	T: Quercetin (150 mg/d)<br>C: PLA<br>RCT (crossover), 42 d (35 d washout phase)	Germany<br>Mixed	Overweight/obese<br>45.1 ± 10.5	Plasma<br>ELISA	Protein levels (pg/mL)<br><br>T: Quercetin (*93*)<br>B: 2.62 ± 2.40; A: 2.37 ± 2.45<br><br>C: PLA (*93*)<br>B: 2.48 ± 2.37; A: 2.40 ± 2.50<br><br>T - C	 <br><br>91%; >100%<br><br><br>95%; >100%	 <br>(*p*<0.01)<br>-9.5%<br><br>(NS)<br>-3.22%<br><br>-0.17 pg/mL (NS)	No significant effect on the levels of TNF-α.<br>No effects on body weight.
[115]	T: Curcuminoids (1 g/d; curcumin, demethoxycurcumin, and bisdemethoxycurcumin)<br>C: PLA<br>RCT (crossover), 30 d (15 d washout phase)	Iran<br>NR	Obese<br>18 – 65	Serum<br>Chemiluminescent immunoassay	Protein levels (pg/mL)<br><br>*Phase 1*<br>T: Curcuminoids (*15*)<br>B: 2.63 ± 2.81; A: 1.50 ± 0.69	 <br><br><br>>100%; 46%	 <br><br>(NS)<br>-43%	No effects on the levels of TNF-α.<br>Effects on body weight not reported.

					C: PLA (*15*)<br>B: 2.72 ± 1.17; A: 0.92 ± 0.29<br>T – C<br>*Phase 2*<br>T: Curcuminoids (*15*)<br>B: 1.29 ± 0.92; A: 1.09 ± 0.46<br>C: PLA (*15*)<br>B: 1.33 ± 0.64; A: 1.52 ± 0.82<br>T - C	43%; 31%<br>71%; 42%<br>48%; 54%	(NS)<br>-66%<br>+0.67 pg/mL (NS)<br>(NS)<br>-15.5%<br>(NS)<br>+14.2%<br>-0.39 pg/mL (NS)	
[116]	T: Curcumin (500 mg/d) or Nano-curcumin (80 mg/d)<br>C: PLA<br>RCT (parallel), 60 d	Iran<br>NR	Overweight cardiac patients<br>T (Curcumin):<br>55.3 ± 7.1<br>T (Nano-curcumin): 56.2 ± 6.9<br>C: 54.1 ± 5.2	Serum<br>ELISA	Protein levels (pg/mL)<br>T: Curcumin (*30*)<br>B: 19.61 ± 4.72; A: 16.01 ± 3.52<br>T: Nano-curcumin (*30*)<br>B: 18.95 ± 4.09; A: 14.06 ± 3.27<br>C: PLA (*30*):<br>B: 19.11 ± 4.23; A: 19.02 ± 4.1	24%; 22%<br>21%; 23%<br>22%; 21%	(p<0.05)<br>-18.3%<br>(p<0.05)<br>-25.8%<br>(NS)<br>-0.47%	Significant reduction of TNF-α levels.<br>No effects on body weight.

					T – C (Curcumin)<br><br>T – C (Nano-curcumin)		-3.5 pg/mL (*p*<0.01)<br><br>-4.8 pg/mL (*p*<0.01)	
[117]	T: Cocoa extract (1.4 g/d, 645 mg of polyphenols) + 15 % energy restricted diet<br>C: PLA + 15% energy restricted diet<br>RCT (parallel), 28 d	Spain<br>Mixed	Overweight/obese<br>57.3 ± 5.2	Plasma<br>ELISA	Protein levels (pg/mL)<br><br>T: Cocoa extract (*23*)<br>B: 1.31 ± 0.60; A: 1.13 ± 0.50<br><br>C: PLA (*24*)<br>B: 1.28 ± 0.63; A: 1.09 ± 0.49<br><br>T - C	 <br><br>46% 44%<br><br><br>49% 45%	 <br>(*p*<0.05)<br>-13.7%<br><br>(NS)<br>-14.8%<br><br>+0.01 pg/mL (NS)	No significant effect on TNF-*α* levels.<br>No effects on body weight
[118]	T: Grape extract capsules (≈ 70 mg mix polyphenols) or Grape extract + Resveratrol capsules (+8 mg Resveratrol)<br>C: PLA<br>RCT (parallel), 1 capsule/d 180 d, 2 capsules/d for another 180 d	Spain<br>Mixed	Overweight/obese with CAD<br>58 ± 9<br>59 ± 10<br>60 ± 12	Serum<br>ELISA	Protein levels (pg/mL)<br><br>T: Grape extract (*25*)<br>B: 14.2 ± 8.7;<br>A180d: 14.4 ± 8.6;<br>A365d: 14.7 ± 9.9;<br><br>T: Grape extract + Resveratrol (*25*)<br>B: 14.9 ± 8.6;	 <br><br>61%<br>60%<br>67%<br><br><br>58%	 <br><br>(NS)<br>+1.4%<br>+3.5%<br><br><br>(NS)	No significant effect on TNF-*α* levels.<br>No changes in body weight.

					A180d: 14.4 ± 8.6;<br>A365d: 13.8 ± 8.2;<br><br>C: PLA (*25*)<br>B: 13.7 ± 8.6;<br>A180d: 14.5 ± 9.2;<br>A365d: 15.2 ± 7.0;<br><br>T – C (Grape extract, 180d)<br>T – C (Grape extract, 365d)<br><br>T – C (Grape extract + Resveratrol, 180d)<br>T – C (Grape extract + Resveratrol, 365d)	60%<br>59%<br><br><br>63%<br>63%<br>46%	-3.3%<br>-7.3%<br><br><br>(NS)<br>+6%<br>+11%<br><br>-0.6 pg/mL (NS)<br>-1.0 pg/mL (NS)<br><br>-1.3 pg/mL (NS)<br><br>-2.6 pg/mL (NS)	

[119]	T: Grape extract capsules (≈ 70 mg mix polyphenols) or Grape extract + Resveratrol capsules (+8 mg Resveratrol);<br>C: Placebo (PLA);<br>RCT (parallel), 1 capsule/d 180 d, 2 capsules/d for another 180 d	Spain<br>Men	Overweight/obese with CAD, T2DM, Hypertension<br>57 ± 10<br>60 ± 10<br>63 ± 12	Serum<br>ELISA	Protein levels (pg/mL)<br>T: Grape extract (*13*)<br>B: 12.1 ± 6.6; A365d: 11.9 ± 5.1;<br>T: Grape extract + Resveratrol (*13*)<br>B: 11.7 ± 5.5; A365d: 10.7 ± 5.8;<br>C: PLA (*9*)<br>B: 12.5 ± 6.4; A365d: 13.9 ± 6.9;<br>T – C (Grape extract)<br>T – C (Grape extract + Resveratrol)	55% 43%<br>47% 54%<br>51% 50%	(NS)<br>-1.6%<br>(NS)<br>-8.5%<br>(NS)<br>+11.2%<br>-1.6 pg/mL (NS)<br>-2.4 pg/mL (NS)	No significant effect on TNF-α level.<br>No changes in body weight with any of the two treatments.<br>In the Grape extract + Resveratrol group down-regulation of *TNF-α* gene expression but not of protein release in PBMCs.
[120]	T: Freeze-dried Grape Powder (46 g/day, 15.6 mg /d of polyphenols)<br>C: PLA<br>RTC (crossover), 21 d (15 d washout phase)	USA<br>Mixed	Obese<br>20-60	Plasma<br>ELISA	Protein levels (pg/mL)<br>T: Grape powder (*24*)<br>B: 3.93 ± 1.92; A: 3.75 ± 2.18<br>C: PLA (*24*)<br>B: 3.81 ± 2.05; A: 3.76 ± 2.00	49% 58%<br>54% 53%	(NS)<br>-4.5%,<br>(NS)<br>1.3%	No significant effects on the levels of TNF-α.<br>No effect on body weight.

					T – C		-0.13 pg/mL (NS)	
[121]	T: Grape powder (60 g/d, rich in polyphenols ~300 mg/d; mostly anthocyanins, flavonols)<br><br>C: PLA<br><br>RCT (crossover), 2-phases: post-prandial and 28 d treatment	USA<br><br>Mixed	Obese<br><br>48.6 ± 15.4	Plasma<br><br>ELISA	Protein levels (pg/mL)<br><br>*Post-prandial (5 h)*<br><br>T: Grape powder (*20*)<br><br>B: 1.5 ± 0.6; A: 1.3 ± 0.5<br><br>C: PLA (*20*)<br><br>B: 1.4 ± 0.4; A: 1.3 ± 0.6<br><br>T – C<br><br>*28 d*<br><br>T: Grape powder (*20*)<br><br>B: 1.5 ± 0.6; A: 1.3 ± 0.6<br><br>C: PLA (*20*)<br>B: 1.4 ± 0.4, A: 1.4 ± 0.6<br><br>T – C	40%, 39%<br><br>29%, 46%<br><br>40%, 46%<br><br>29%, 43%	(NS)<br><br>-13.3%<br><br>(NS)<br><br>-7.1%<br><br>-0.1 pg/mL (NS)<br><br>(NS)<br><br>-13.3%<br><br>(NS)<br><br>0%<br><br>-0.2 pg/mL (NS)	No significant effects on the levels of TNF-α.<br><br>The effects on body weight were not reported.
[122]	T: Grape pomace extract + Omija fruit (342.5 mg/d + 57.5 mg/d for low dose	Korea<br><br>Mixed	Overweight/obese<br><br>30–70	Plasma<br><br>ELISA	Protein levels (pg/mL)			Significant but rather small reduction of TNF- α levels with high dose of Grape

	group and 685 mg/d + 11 5 mg/d for high dose group)<br>C: PLA<br>RCT (parallel), 70 d				T: Grape pomace extract-high dose (*26*)<br>B: 5.56 ± 2.96; A: 3.56 ± 2.79<br>T: Grape pomace extract-low dose (*26*)<br>B: 4.56 ± 2.66; A: 3.78 ± 2.47<br>C: PLA (*24*)<br>B: 4.87 ± 2.28; A: 4.24 ± 2.41<br>T – C (high dose)<br>T – C (low dose)	53% 78%<br>58% 65%<br>47% 57%	(p<0.05)<br>-36%<br>(NS)<br>-17%<br>(NS)<br>-13%<br>-1.37 pg/mL (p<0.05)<br>-0.15 pg/mL (NS)	pomace extract + Omija fruit. No effect on body weight.
[123]	T: Grape seed extract (300 mg/d containing 85% polyphenols) + LCD<br>C: PLA + LCD<br>RCT (parallel), 84 d	Iran<br>Mixed	Overweight/obese<br>T: 35.0 ± 1.9<br>C: 33.5 ± 2.0	Serum<br>ELISA	Protein levels (pg/mL)<br>T: Grape seed extract (*20*)<br>B: 24.3 ± 3.55 A: 17.9 ± 4.6<br>C: PLA (*20*)<br>B: 17.6 ± 0.74 A: 23.1 ± 3.48<br>T – C	15% 26%<br>4% 15%	(p<0.01)<br>-26.3%,<br>(p<0.01)<br>+31.25%	Significant reduction of TNF-α levels.<br>Significant decreased in body weight.

							-11.9 pg/mL<br>(p=0.0001)	
[124]	T: Pomegranate peel extract (250 mg/d,<br>C: Placebo;<br>RTC (parallel), 56 d	Bosnia and Herzegovina<br>Mixed	Overweight, T2DM<br>T: 57.9 ± 6.1<br>C: 56.9 ± 6.7	Plasma<br>ELISA	Protein levels (pg/mL)<br>T: Pomegranate peel extract (*30*)<br>B: 0.17 (0.8 – 0.27);<br>A: 0.13 (0.6 – 0.23)<br>C: PLA (*14*)<br>B: 0.18 (0.3 – 0.34)<br>A: 0.19 (0.5 – 0.33)<br>T - C	ND<br>ND<br>ND<br>ND	(p<0.05)<br>-23%<br>(NS)<br>-5%<br>-0.05 pg/mL (p<0.05)	Values estimated from figures (median (IQR)). Significant but rather small reduction of the levels of TNF-α with T.<br>Effects on body weight not reported.
[125]	T: Freeze-dried strawberries (50 g/d)<br>C: PLA<br>RCT (crossover), 84 d (14 d washout phase)	USA<br>Mixed	Obese, knee OA<br>57 ± 7	Serum<br>ELISA	Protein levels (pg/mL)<br>T: Freeze-dried strawberries (*8*)<br>B: 5.5 ± 1.5; A: 2.8 ± 2.6<br>C: PLA (*8*)<br>B: 5.5 ± 1.5; A: 5.2 ± 2.1<br>T – C	27% 93%<br>27% 40%	(p<0.05)<br>-49%<br>(NS)<br>5%<br>-2.7 pg/mL (p=0.02)	Significant reduction of the levels of TNF-α with T.<br>No effects on body weight.

[126]	T: Frozen red raspberry (250 g/d containing 11.5 g fiber and 343 mg polyphenols, mostly anthocyanins, ellagitannins)<br>C: PLA<br>RCT (crossover), 2 phases: post-prandial and 28 d	USA<br>Mixed	Obese, T2DM<br>54.0 ± 4.2	Serum<br>ELISA	Protein levels (pg/mL)<br>*Post-prandial (4 h)*<br>T: Frozen red raspberry (*25*)<br>B: 6.3 ± 12.5; A: 9.3 ± 32.5<br>C: PLA (*25*)<br>B: 4.7 ± 7.5; A: 18.7 ± 37.5<br>T – C<br>*28 d*<br>T: Frozen red raspberry (*22*)<br>B: 5.5 ± 7.0; A: 3.2 ± 9.8<br>C: PLA (*22*)<br>B: 5.5 ± 11.7; A: 5.3 ± 15.5<br>T – C	>100%<br>>100%<br>>100%<br>>100%	(NS)<br>+47%<br>(NS)<br>+297%<br>-11.0 pg/mL ($p<0.05$)<br>($p<0.05$)<br>-42%<br>(NS)<br>-4%<br>-2.1 pg/mL ($p<0.05$)	Significant reduction of the levels of TNF-α with T.<br>Effect on body weight not reported.
[127]	T: Black soybean testa extracts (2.5 g/d rich in anthocyanins)<br>C: PLA (starch 2.5 g/d)	Korea<br>Mixed	Overweight/obese<br>T: 30.30 ± 9.42<br>C: 30.88 ± 9.18	Plasma<br>ELISA	Protein levels (pg/mL)<br>T: Soybean extract (*32*)<br>B: 15.96 ± 3.73;	23%	($p<0.05$)	No significant effects on the levels of TNF-α.<br>No effect on body weight.

	RCT (parallel), 56 d				A: 15.17 ± 2.97<br><br>C: PLA (*31*)<br>B: 43.01 ± 19.49;<br>A: 39.63 ± 12.69<br><br>T – C	19%<br><br>45%<br>32%	-5%<br><br>(*p*<0.05)<br>-8%<br><br><br>+2.61 pg/mL (NS)	
Other phytochemical-containing products								
[128]	T: Fruit + Vegetable concentrate powder (6 capsules/d, β-carotene, α-tocopherol, vitamin C, folate, polyphenols)<br>C: PLA<br>RCT (parallel), 56 d	UK<br>Mixed	Overweight/obese<br>T: 61.4 ± 1.5<br>C: 57.9 ± 1.4	Plasma<br>ELISA	Protein levels (pg/mL)<br><br>T: Fruit + Vegetable (*28*)<br>B: 1.04 (0.87, 1.41)<br>A: 1.02 (0.55, 1,41)<br><br>C: PLA (*28*)<br>B: 1.07 (0.79, 1.22)<br>A: 0.94 (0.82, 1.26)<br><br>T - C<br><br>*Subgroup (CRP ≥3.0 mg/mL)*<br><br>T: Fruit + Vegetable (*16*)	 <br><br><br>ND<br><br><br><br>ND	 <br><br>(*p*=0.037)<br>-2%<br><br><br>(NS)<br>-12%<br><br>+0.11 pg/mL (NS)	For all subjects:<br>No reduction in TNF-α levels.<br>No effects on body weight.<br><br>For subjects with CRP (≥3.0 mg/mL):<br>Reduction in TNF-α levels.<br>No effects on body weight.

					B: 1.13 (0.95, 1.52)<br>A: 0.95 (0.70, 1.40)<br><br>C: PLA (*15*)<br>B: 1.07 (0.79, 1.30)<br>A: 0.96 (0.87, 1.26)<br><br>T - C	 ND<br><br><br>ND 	(*p*=0.07)<br>-16%<br><br><br>(NS)<br>-10%<br><br>-0.07 pg/mL (*p*=0.035)	
[129]	T: Juice Plus+ Premium® (6 capsules/d, 2.91 mg carotene, 18.7 mg Vitamin E, 159 mg Vitamin C, 318 µg folate, 6.1 mg lutein, 1 mg lycopene and 0.15 mg astaxanthin)<br>C: PLA<br>RCT (crossover), 112 d (28 d washout phase)	Spain<br>Mixed	Mixed subjects (BMI between 18.5 and 35)<br>34 ± 11	Serum<br>ELISA	Protein levels (pg/mL)<br><br>T: Juice Plus (*92*)<br>B: 5.82 ± 1.14; A: 5.22 ± 1.21<br><br>C: PLA (*92*)<br>B: 5.69 ± 1.02; A: 5.30 ± 0.87<br><br>T - C	 <br><br>18%; 16%<br><br><br>19%; 23% 	 <br>(*p*<0.01)<br>-10%<br><br>(*p*<0.05)<br>-7%<br><br>-0.21 pg/mL (NS)	No reduction in TNF-*α* levels.<br>No effects on body weight.
[130]	T: Melatonin (6 mg/d) + LCD<br>C: PLA + LCD<br>RCT (parallel), 40 d	Iran<br>Women	Obese<br>T: 33.8 ± 6.9<br>C: 34.8 ± 7.3	Serum<br>ELISA	Protein levels (pg/mL)<br><br>T: Melatonin (22)<br>B: 3.52 ± 0.72; A: 1.73 ± 0.07<br><br>C: PLA (22)	 <br><br>20%; 4%<br><br>18%; 19%	 <br>(*p*<0.05)<br>-50%<br><br>(NS)	Reduction of TNF-*α* levels.<br>No effect on body weight.

					B: 2.82 ± 0.52; A: 2.01 ± 0.38<br><br>T - C		-29%<br><br>-0.98 pg/mL (*p*=0.02)	
[131]	T: Garlic tablets (1000 mg/d containing 2.5 mg of allicin),<br>C: PLA<br>RCT (parallel), 84 d	Iran<br>Women	Overweight/obese,<br>knee OA,<br>58.7 ± 7.4	Serum<br>NR	Protein levels (pg/mL)<br><br>T: Garlic tablets (39)<br>B: 62.8 ± 73.3; A: 64.1 ± 73.5<br><br>C: PLA (37)<br>B: 61.8 ± 64.7; A: 49.1 ± 40.5<br><br>T - C	 <br><br>117%; 115%<br><br><br>105%; 82%	 <br>(NS)<br>+2%<br><br>(NS)<br>-21%<br><br>+14.0 pg/mL (NS)	No effect on the levels of TNF-*α*.<br>No effects on body weight.

Legend: [a]: Most values presented as the mean ± standard deviation (SD), some data indicated as Median (Inter Quartile Range, IQR); [b]:(*N*): Sample size per group; [c]: CV%: Coefficient of variation in percentage estimated for each analyzed subgroup ((CV%) = (Standard Deviation/Mean) × 100); B: Before intervention; A: After intervention; [d]:Difference in % before and after intervention; T: Treatment; C: Control; T – C[e]: effect size calculated as the difference between the T and the C group (pg/mL); BMI: Body mass index; CAD: Coronary artery disease; LCD: Low calories diet; ND: Not determined; NR: Not reported; NS: Not significant; OA: Osteoarthritis; PBMCs: Peripheral blood mononuclear cells; PLA: Placebo; RCT: Randomized clinical trial; T2DM: type 2 diabetes mellitus; TNF-*α*: tumor necrosis factor alpha.

Table S6. Reported changes in the circulatory levels of human TNF-α following dietary intervention with foods rich in mixed bioactive phytochemicals.

	Intervention	Sample population		TNF-α levels				
Ref.	Tested product/main compounds<br>Study design	Country<br>Sex	Body weight phenotype and comorbidities groups<br>Age (y)[a]	Type of Sample<br>Method	Results per group[a] (*N*)[b]	CV%[c]	(Sig)<br>Effect<br>(% A *vs* B[d] per group<br>T – C[e] pg/mL)	General message conveyed by the article.
[132]	T: Juçara pulp (5 g/d; phenolics, flavonoids)<br>C: PLA<br>RCT (parallel), 42 d	Brazil<br>Mixed	Obese<br>T: 45.1 ± 3.4<br>C: 45.8 ± 2.6	Supernatant of isolated PBMC<br>ELISA	Protein levels (pg/mg of proteins)<br><br>T: Jucara pulp (*10*)<br>B: 650 ± 200; A: 300 ± 150<br><br>C: PLA (*10*)<br>B: 780 ± 200; A: 300 ± 100<br><br>T - C	 <br><br>30% 50%<br><br><br>25% 33%	 <br>(NS)<br>-54%<br><br>(NS)<br>-61%<br><br>+130 (NS)	No significant effect on the expression and release of TNF-α by isolated PBMC.<br>Effect on body weight not reported.
[133]	T: Queen Garnet plums juice (250 mL/d; anthocyanins, quercetin)<br>C: PLA (Raspberry cordial)<br>RCT (parallel), 84 d	Australia<br>Mixed	Overweight/obese with Hypertension<br>20-60	Plasma<br>ELISA	Protein levels (pg/mL)<br><br>T: QG juice (*15*)<br>B: 5.6 ± 2.6; A: 3.4 ± 3.4<br><br>C: Raspberry cordial (*14*)<br>B: 7.2 ± 5.9; A: 7.0 ± 3.2<br><br>T - C	 <br><br>46% 100%<br><br><br>82% 46%	 <br>($p<0.05$)<br>-39%<br><br>(NS)<br>-3%<br><br>-2.0 pg/mL ($p<0.05$)	Significant reduction of the levels of TNF-α with T.<br>No effect on body weight.
[134]	T: Tart cherry juice (240 mL/d; anthocyanins, flavonoids, phenolics)<br>C: PLA<br>RCT (crossover), 28 d (14 d washout phase)	USA<br>Mixed	Overweight/obese<br>38.1 ± 12.5	Plasma<br>ELISA	Protein levels (pg/mL)<br><br>T: Tart cherry juice (*10*)<br>B: 5.83 ± 1.14; A: 5.53 ± 0.94<br><br>C: PLA (*10*)<br>B: 5.56 ± 1.07; A: 5.47 ± 0.94<br><br>T – C	 <br><br>19% 17%<br><br><br>19% 17%	 <br>(NS)<br>-5%<br><br>(NS)<br>-1%<br><br>-0.21 pg/mL (NS)	No significant effect on the levels of TNF-α.<br>No effect on body weight.
[135]	T: Tomato juice (330 mL/day; carotenoids, phytosterols, phenolics, flavonoids, anthocaynins)<br>C: PLA	Iran<br>Women	Overweight/obese<br>T: 23.3 ± 3.6<br>C: 23.2 ± 2.9	Serum<br>ELISA	Protein levels (pg/ml)<br><br>T: Tomato juice (*53*)<br>B: 73.2 ± 92.45; A: NR	ND	ND	Authors did not present TNF- α values before and (or) after the treatment but they reported a significant reduction of TNF- α levels, especially in the

	RCT (parallel), 20 d				C: PLA (*51*)<br>B: 66.7 ± 84.5; A: NR<br><br>T – C<br><br>*Subgroup overweight*<br>T: Tomato juice (*43*)<br>B: NR; A: NR<br><br>C: PLA (*42*)<br>B: NR; A: NR<br><br>T – C<br><br>*Subgroup Obese*<br>T: Tomato juice (*10*)<br>B: NR; A: NR<br><br>C: PLA (*9*):<br>B: NR; A: NR<br><br>T – C	ND<br><br><br><br><br>ND<br><br>ND<br><br><br><br><br>ND<br><br>ND	ND<br><br>-8.3 pg/mL (*p*<0.05)<br><br><br>ND<br><br>ND<br><br>-5.3 pg/mL (*p*<0.05)<br><br><br>ND<br><br>ND<br><br>+21.3 pg/mL (NS)	overweight subgroup. No effect on the levels of TNF- *α* in obese subgroup.<br>Effect on body weight not reported.
[136]	T: Red sorghum flaked biscuits (45 g/d; carotenoids, phytosterols, phenolics, flavonoids) + LCD;<br>C: Wheat biscuits (45 g/d) + LCD<br>RCT (parallel), 84 d	Australia<br>Mixed	Overweight/mildly obese<br>T: 48.1 ± 10.3<br>C: 48.6 ± 11.4	NR	Protein levels (pg/mL)<br><br>T: Sorghum biscuits (*26*)<br>B: 3.2 ± 1.2; A: NR<br><br>C: Wheat biscuits (*30*)<br>B: 2.9 ± 1.3; A: NR<br><br>T – C	 <br><br>37% ND<br><br><br>45% ND	 <br><br>ND<br><br><br>ND<br><br>-0.3 pg/mL (NS)	No significant effect on the levels of TNF-*α*.<br>No effect on body weight.

Legend: [a]: Values are presented as the mean ± SD; [b]:(*N*): Sample size per group; [c]: CV%: Coefficient of variation in percentage estimated for each analyzed subgroup ((CV%) = (Standard Deviation/Mean) × 100); B: Before intervention; A: After intervention; [d]:Difference in % before and after intervention; T: Treatment; C: Control; T – C[e]: effect size calculated as the difference between the T and the C group (pg/mL); LCD: Low calorie diet; ND: Not determined; NR: Not reported; NS: Not significant; PBMCs: peripheral blood mononuclear cells; PLA: Placebo; QC: Queen Garnet; RCT: Randomized clinical trial; TNF-*α*: tumor necrosis factor alpha.

Table S7. Circulating levels of TNF-α across different genotypes for various SNPs located within the regulatory region of the *TNFα* gene.

	Genetic variant	Sample population			Genotype Protein levels[a] (pg/mL) (CV%)[c]				
Ref	SNP	Country Sex	Phenotype group Age[a] (y)	*N*[b]	Sample Method	Homozygous reference (*N*)[d]	Heterozygous (*N*)[d]	Homozygous variant (*N*)[d]	(Sig) Effect (pg/mL)[e]
[137]	*(-308 G/A) rs1800629*	Brazil Mixed	High cardiometabolic risk (pre-diabetes or MetS) 56.5 ± 11.6	138	Plasma Immuno-enzyme chemi-luminescent assay	GG (107) 0.11 (0.09 – 0.13)[f] (ND)	GA+AA (28) 0.11 (0.09 – 0.14)[f] (ND)		(NR) (NC)
[139]	*(-308 G/A) rs1800629*	Spain Mixed	Obese patients 45.8 ± 16.4	128	Unclear ELISA	GG (91) 1.4 ± 0.6 (42.9%)	GA+AA (37) 1.6 ± 1.3 (81.3%)		(NR) +0.2
				133		GG (106) 2.01 ± 1.8 (89.6%)	GA+AA (27) 1.8 ± 2.1 (>100%)		(NR) +0.2
[41]	*(-308 G/A) rs1800629*	Brazil Mixed	Mix (normal, overweight, T2DM) 56.0 (12.0)[f] 53.0 (18.0)[f]	264	Plasma Cytometric bed array	GG (125) 0.095 (0.055)[f] (ND)	GA (38) 0.090 (0.063)[f] (ND)	AA (1) 0.048 (ND)	(NS) -0.005, -0.047
			T2DM 56.0 (12.0)[f]	102		GG (45) 0.095 (0.045)[f] (ND)	GA (44) 0.085 (0.044)[f] (ND)	AA (1) 0.048 (ND)	(NS) -0.01, -0.047
[140]	*(-308 G/A) rs1800629*	Brazil Mixed	General population 38.4 ± 12.5, 38.2 ± 12.1	280	Plasma Multiplex	GG (226) 4.9 ± 2.0 (41%)	GA+AA (54) 4.8 ± 1.8 (38%)		(NS) -0.1
	(-857C/T) rs1799724		General population 37.8 ± 12.2, 40.7 ± 12.6			CC (227) 4.9 ± 2.0 (41%)	CT+TT (52) 4.6 ± 2.0 (43%)		(NS) -0.2
	(-1031T/C) rs17999964		General population 37.9 ± 12.7, 38.9 ± 11.9			TT (152) 4.9 ± 1.8 (37%)	TC+CC (128) 4.6 ± 1.9 (41%)		(NS) -0.3
	(-238G/A) rs361525		General population 38.2 ± 12.6,			GG (243) 4.8 ± 1.9	GA+AA (37) 5.5 ± 2.1		(*p*=0.033) +0.7

			39.5 ± 10.9			(40%)	(38%)		
[141]	*(-308 G/A)*<br>*rs1800629*	Poland<br>Women	MetS<br>53.0 ± 5.0	253	Serum<br>ELISA	GG (191)<br>3.5 ± 14.9<br>(>100%)	GA (54)<br>3.2 ± 8.4<br>(>100%)	AA (8)<br>4.0 ± 4.4<br>(>100%)	(NS)<br>-0.3, +0.5
				90		GG (65)<br>4.2 ± 9.2<br>(>100%)	GA (25)<br>3.4 ± 6.1<br>(>100%)	NR	(NS)<br>-0.8
[142]	*(-308 G/A)*<br>*rs1800629*	India<br>Mixed	NAlb<br>53.9 ± 10.9	196	Plasma<br>ELISA	GG (121)<br>56.2 (48.7 – 65.9)<br>(ND)	GA (47)<br>(NR)	AA (28)<br>99.1 (46.2 – 143.6) (ND)	(NR)<br>+42.9
[42]	*(-308 G/A)*<br>*rs1800629*	Pakistan<br>Mixed	Healthy normoweight<br>46.5 ± 8.2	200	Serum<br>ELISA	GG (102)<br>14.0 (5.5 – 20.5)[f]<br>(ND)	GA (76)<br>8.0 (6.0 – 13.5)[f]<br>(ND)	AA (22)<br>18.0 (7.0 – 83.0)[f]<br>(ND)	(NS)<br>-6.0<br>+4.0
			MetS overweight<br>47.0 ± 8.0	224		GG (100)<br>13.0 (7.0 – 43.0)[f]<br>(ND)	GA (101)<br>30.0 (12.0 – 60.0)[f]<br>(ND)	AA (23)<br>68.0 (26.0 – 146)[f]<br>(ND)	(*p*=0.060)<br>+17.0<br>(*p*=0.001)<br>+38.0
[143]	*(-308 G/A)*<br>*rs1800629*	Iraq<br>Mixed	Patients with vitiligo<br>33.0 ± 12.7<br>28.8 ± 12.4	40	Serum<br>ELISA	GG (27)<br>6.9 ± 4.8<br>(70%)	GA (11)<br>5.8 ± 2.9<br>(50%)	AA (2)<br>4.1 ± 0.6<br>(15%)	(NR)<br>-1.1<br>-2.5
				80		GG (46)<br>13.5 ± 16.5<br>(>100%)	GA (21)<br>11.5 ± 7.8<br>(68%)	AA (13)<br>12.9 ± 14.5<br>(>100%)	(NS)<br>-2.0<br>-0.6
[144]	*(-308 G/A)*<br>*rs1800629*	Austria<br>Mixed	Patients with AAA<br>69.7 ± 9.6<br>70.5 ± 7.0	216	Plasma<br>ELISA	GG (115)<br>28.1 (31.6)[f]<br>(>100%)	GA+AA (101)<br>78.2 (93.3)[f]<br>(>100%)		(*p*=0.045)<br>+50.1

Legend: [a]: Data are shown as the mean value ± SD unless otherwise indicated; [b]: Sample size of the full genotyped group; [c]: CV%: Coefficient of variation (%) ((CV%) = (Standard Deviation/Mean) × 100); [d]: Sample size of each of the genotype subgroups; [e]: Change (effect size) attributed to a different genotype estimated as the difference between heterozygous – homozygous reference and between homozygous variant – homozygous reference; [f]: median (IQR); A: Adenine; AAA: Abdominal aortic aneurism; C: Cytosine; G: Guanine; MetS: Metabolic syndrome; NAlb: Normoalbuminuria; NC: No change; ND: Not determined; NR: Not reported; NS: Not significant; T: Thymine; T2DM: Type 2 diabetes mellitus.

Table S8. Circulating levels of TNF-α across different genotypes for different SNPs located in various genes related to obesity, inflammatory and metabolic disorders.

	SNP description		Sample population			Genotype<br>Protein levels[a] (pg/mL)<br>(CV%)[c]				
Ref	Gen/<br>SNP	Metabolic association	Country<br>Sex	Phenotype group<br>Age[a] (y)	*N*[b]	Sample<br>Method	Homozygous reference (*N*[d])	Heterozygous (*N*[d])	Homozygous variant (*N*[d])	(Sig)<br>Effect (pg/mL)[e]
[145]	*MTHFR*/<br>C677T	Body fat accumulation<br>Inflammatory response	Brazil<br>Women	Overweight/<br>Obese<br>44.3 ± 9.3	24	Serum<br>Chemi-luminiscent solid-phase system	CC (8)<br>5.8 ± 8.3<br>(>100%)	CT (8)<br>9.5 ± 8.6<br>(90.5%)	TT (8)<br>3.1 ± 2.3<br>(74.2%)	(NR)<br>+3.7<br>-2.7
				Overweight/<br>Obese<br>44.8 ± 12.5	24		CC (8)<br>7.7 ± 12.4<br>(>100%)	CT (8)<br>5.0 ± 4.6<br>(92.0%)	TT (8)<br>6.8 ± 5.2<br>(76.5%)	(NR)<br>-2.7<br>-0.9
[146]	*PPARD*/<br>-87T>C	Plasma lipid profile	China<br>Mixed	T2DM<br>63.5 ± 10.5	286	Plasma<br>ELISA	TT (136)<br>12.8 ± 7.4<br>(57.8%)	TC (128)<br>11.1 ± 4.8<br>(43.2%)	CC (22)<br>9.7 ± 4.2<br>(43.3%)	(p<0.05)<br>-1.7<br>-3.1
				Normal glucose<br>64.6 ± 12.6	158		TT (75)<br>11.0 ± 6.7<br>(61.0%)	TC (72)<br>11.1 ± 7.5<br>(67.6%)	CC (11)<br>12.1 ± 8.8<br>(72.7%)	(NS)<br>+0.1<br>+1.1
[147]	*VDR*/<br>FOK1	Bone mineral density	Iran<br>Women	Obese<br>37.8 ± 11.1,<br>42.0 ± 13.1	264	Serum<br>(NR)	FF (184)<br>5.1 ± 6.4<br>(>100%)	Ff + ff (80)<br>12.6 ± 26.6<br>(>100%)		(p=0.01)<br>+7.5
[148]	*CLOCK*/<br>rs3749474	Circadian rhythm and energy balance	USA<br>Mixed	GOLDN population (overweight)<br>48.5 ± 16.3	1110	Plasma<br>Enzyme-linked immune sorbent assay	CC (427)<br>3.3 ± 8.3<br>(>100%)	TT+CC (666)<br>3.6 ± 6.7<br>(>100%)		(NS)<br>+0.3
	CLOCK/<br>rs4580704						CC (481)<br>3.6 ± 7.0<br>(>100%)	GG+CG (619)<br>3.3 ± 8.0<br>(>100%)		(NS)<br>-0.3
	CLOCK/<br>rs1801260						AG+AA (914)<br>3.5 ± 12.7<br>(>100%)		GG (183)<br>3.1 ± 5.7<br>(>100%)	(NS)<br>-0.4
[149]	*IL6*/<br>rs1800795	Inflammation, lipid metabolism, energy balance	Poland<br>Women<br>Post-menopause	Central obesity + ≥1 feature MetS<br>60.5	144	Serum<br>ELISA	GG (36)<br>8.2 ± 3.6<br>(43.9%)	GC+CC (108)<br>11.3 ± 7.3<br>(64.6%)		(NS)<br>+3.1
[150]	*UCP3*/	Energy balance	Spain		64	Unclear	CC (NR)	CT+TT (NR)		(NR)

	-55CT		Mixed	Obese non diabetic<br>NR			3.9 ± 2.7<br>(69.2%)	2.3 ± 2.7<br>(>100%)		-1.6
					67		CC (NR)<br>4.3 ± 2.8<br>(65.1%)	CT+TT (NR)<br>4.2 ± 2.9<br>(69.0%)		(NR)<br>-0.1
[151]	*FTO*/<br>rs9939609	Associated with obesity	Spain<br>NR	Obese<br>(BMI>40)<br>50.0 (17.3)[f]<br>48.9 (16.2)[f]	129	Serum<br>ELISA	TT (41)<br>7.0 (6.3)[f]<br>(ND)	AT+AA (88)<br>6.3 (4.4)[f]<br>(ND)		(NS)<br>-0.7
[152]	*TLR1*/<br>A743G &<br>T1805G	Immune response to infections and vaccination. Levels of cytokines and age.	Germany/<br>Italy<br>Mixed	Elderly sample population<br>70.0<br>70.5<br>92.8	187	Plasma<br>Multiplex<br>beads	*TLR1* –<br>AA/GG (66-76)<br>≈90 ± 60*<br>(44.4%)	(all) *TLR1* +<br>AG+GG/TG+TT (88-89)<br>≈80 ± 70*<br>(87.5%)		(NS)<br>-10
[153]	*CAT*/<br>rs769214	Glucose homeostasis; malnutrition	France<br>Mixed	Malnourished elderly<br>83.3 ± 6.8	33	Serum<br>Bio-Plex	GG (8)<br>6.9 ± 6.8<br>(98.6%)	GA (17)<br>3.7 ± 0.8<br>(21.6%)	AA (8)<br>3.0 ± 1.1<br>(36.7%)	(*p*<0.001)<br>-3.2<br>-3.9
[154]	*TGFB*/<br>rs1800471	Fibrosis; heart failure	Poland<br>Mixed	Heart failure patients<br>63.0 ± 11.0	110	Serum<br>ELISA	CC (88)<br>21 (10 - 31)[f]<br>(ND)	CG (22)<br>14 (2.9 - 21.0)[f]<br>(ND)	NR	(*p*=0.042)<br>-7
[137]	*IL6*/<br>-174 G/C	Adiposity, inflammation and metabolic disturbances	Brazil<br>Mixed	High cardio-metabolic risk (pre-diabetes, MetS)<br>56.5 ± 11.6	138	Plasma<br>Immuno enzyme chemilumi nescent assay	GG (47)<br>0.11<br>(0.09 – 0.14)[f]<br>(ND)	GG+CC (86)<br>0.11<br>(0.09 – 0.13)[f]<br>(ND)		(NS)<br>NC
	ADIPOQ/<br>45 T/G	Hypo-adiponectinemia, risk of T2DM and MetS					TT (94)<br>0.11<br>(0.09 – 0.13)[f]<br>(ND)	TG+GG (37)<br>0.11<br>(0.10 – 0.13)[f]<br>(ND)		(NS)<br>NC
[155]	*ZIP2*/<br>A/G/T<br>rs2234632	Severe carotid artery disease and inflammation	Italy, Poland, Germany, Greece, France<br>Mixed	ZINCAGE population (healthy elderly people)<br>74.9 ± 8.5	1090	Plasma<br>Multiplex<br>bead-based assay	Leu – (535)<br>76.6 ± 43.9<br>(57.3)	Leu + (555)<br>70.5 ± 43.2<br>(61.3)		(*p*=0.011)<br>-6.1
[156]	*TNF-LTA* cluster/<br>rs2229094	Metabolic traits including susceptibility to	Canada<br>Mixed	Healthy, normal	191	Plasma<br>ELISA	TT (108)<br>≈1.9 ± 2.0<br>(>100%)	CT (66)<br>≈1.5 ± 0.5<br>(33.3%)	CC (17)<br>≈1.6 ± 0.5<br>(31.3%)	(NS)<br>-0.4<br>-0.3

		T2DM, MetS, IR, and increased BMI		weight, overweight 30.8 ± 8.7						
[157]	*TMEM182*/ Rs141764639	Strongly associated with WC	Korea Mixed	Normal 48.5 ± 0.30	1314	Serum Bio-Plex	TT (1252) ≈10.0 ± 35.0 (>100%)	TC (62) ≈14.5 ± 32.0 (>100%9	CC (32) (NR)	(*p*<0.05) +4.5
				Central obese 51.4 ± 0.42	827		TT (749) ≈9.5 ± 14.0 (>100%)	TC (78) ≈23.0 ± 26.0 (>100%)	CC (39) (NR)	(*p*<0.001) +13.5
[158]	*TLR2*/ rs4696480	Receptors of bacterial products.	Spain Mixed	BMI < 30 49 ± 15	104	Serum ELISA	Wild type (47) ≈5.3 ± 2.5 (47.2%)	Variant (57) ≈4.2 ± 3.9 (92%)	-	(NS) -1.1
	TLR9/ rs187084						Wild type (65) ≈5.5 ± 3.8 (69%)	Variant (39) ≈4.2 ± 4.2 (100%)	-	(NS) -1.3
	TLR4/ rs4986790						Wild type (99) ≈6.5 ± 3.8 (58.5%)	Variant (5) ≈4.9 ± 2.5 (51%)	-	(NS) -1.6
	TLR2/ rs4696480			BMI > 30 49 ± 14	211		Wild type (89) ≈5.5 ± 4.7 (85%)	Variant (122) 3.8 ± 2.7 (71%)	-	(NS) -1.7
	TLR9/ rs187084						Wild type (131) ≈4.2 ± 2.7 (64%)	Variant (80) ≈4.4 ± 3.3 (75%)	-	(NS) +0.2
	TLR4/ rs4986790						Wild type (202) ≈4.2 ± 2.5 (60%)	Variant (9) ≈4.4 ± 3.1 (70%)	-	(NS) +0.2

Legend: [a]: Data are shown as the mean value ± SD unless otherwise indicated; [b]: Sample size of the full genotyped group; [c]: CV%: Coefficient of variation (%) ((CV%) = (Standard Deviation/Mean) × 100); [d]: Sample size of each of the genotype subgroups; [e]: Change (effect size) attributed to a different genotype estimated as the difference between heterozygous – homozygous reference and between homozygous variant – homozygous reference; [f]: median (IQR); BMI: Body mass index; IR: Insulin resistance; MetS: Metabolic syndrome; NC: No change; ND: Not determined; NR: Not reported; NS: Not significant; T2DM: Type 2 diabetes mellitus; WC: Waist circumference; ≈: Values estimated from bars figure. G: Guanine; A: Adenine; C: Cytosine; T: Thymine. Genes: *MTHFR*: Methylenetetrahydrofolate reductase; *PPARD*: POeroxisome proliferator activated receptor delta; *VDR*: Vitamin D receptor; *CLOCK:* Clock circadian regulator.; *IL6:* Interleukin 6; *UCP3:* Uncoupling protein 3; *FTO:* FTO alpha-ketoglutarate dependent dioxygenase; *TLR:* Toll like receptor; *CAT:* Catalase; *TGFB*: Transforming growth factor; *ADIPOQ:* Adiponectin; *ZIP2*: Zinc transporter protein; *TNF-LTA*: TNF-LTA gene cluster; *LTA*: Lymphotoxin alpha; *TMEM182:* Transmembrane protein 182.

Table S9. Reported changes on the circulatory levels of human TNF-α following intervention with specific drugs in relation with obesity treatment.

	Intervention	Sample population		TNF-α levels				General message conveyed by the article
Ref.	Tested drug<br>Study design	Country<br>Sex	Body weight phenotype and co-morbidities group,<br>Age[a] (y)	Type of Sample<br>Method	Results per group[a] (*N*)[b]	CV%[c] per group	(Sig)<br>Effect<br>(%A *vs* B per group;<br>T – C[e] pg/mL)	
Chemical drugs								
[163]	T: Orlistat (360 mg/d) + LCD<br>C: LCD<br>RCT, 365 d	Turkey<br>Mixed	Obese, T2DM<br>C: 53.2 ± 9.9<br>T: 53.7 ± 9.4	Plasma<br>ELISA	Protein levels (pg/mL)<br>T: Orlistat + LCD (*190*)<br>B: NR<br>A: 13.6 (0.1 – 89.8)[d]<br><br>C: LCD (*186*)<br>B: NR<br>A: 14.1 (0.3 – 96.1)[d]<br><br>T - C	ND	% ND<br><br>-0.5 pg/mL (based only on values after intervention)	Body weight reduction in both C and T groups. Association with lower levels of TNF-α in both groups but slightly lower in the Orlistat group than in the C subjects.<br>Insufficient information on the statistical differences between the two groups and it is not possible to ascribe any clear effect to Orlistat.
[164]	T: Orlistat (NR mg/d) + LCD<br>C: LCD<br>RCT (open-label), 183 d	Greece<br>Women	Obese<br>C: 35.4 ± 9.2<br>T: 38.0 ± 7.1	Serum<br>ELISA	Protein levels (pg/mL)<br>T: Orlistat + LCD (*35*)<br>B: 43 ± 15.9<br>A: 22.3 ± 7.5<br><br>C: LCD (*36*)<br>B: 41.1 ± 12.7<br>A: 32.5 ± 10.3<br><br>T – C	 <br>31%<br>32%<br><br><br>37%<br>34%	(*p*<0.001) (-48%)<br><br>(*p*<0.001) (-21%)<br><br>(-12.1 pg/mL) (*p*<0.001)	Body weight and TNF-α reduction in both groups. Significant differences between T and C and thus, Orlistat potentiated the effect of a LCD in the reduction of TNF-α. It also potentiated the improvements in metabolic and inflammatory dysregulation.
[165]	T: Orlistat (360 mg/d) + LCD<br>C: LCD<br>RCT (open-label), 60 d	Brazil<br>Women	Obese, Hypertensive<br>C: 46.8 ± 5.8<br>T: 47.9 ± 9.3	Serum<br>ELISA	Protein levels (pg/mL)<br>T: Orlistat + LCD (*14*)<br>B: 24.5 ± 13.4<br>A: 9.7 ± 7.0	 <br>55%<br>72%	(*p*<0.05)<br>(-60%)	Reduction of body weight and TNF-α levels in both groups, but no significant differences between T and C. Orlistat did not clearly

					C: LCD (*10*)<br>B: 18.8 ± 7.1<br>A: 8.2 ± 1.2<br><br>T – C	 38%<br>15%	 (*p*<0.05)<br>(-56%)<br><br>-4.2 pg/mL (NS)	improved the effect of a LCD on TNF-α and did not add any further metabolic and/or anti-inflammatory improvements.
[166]	T: Orlistat (120 mg ×3/d)+ L-Carnitine (2 g/d)<br>C: Orlistat (120 mg ×3/d)<br>RCT double-blind controlled, 90 d, 180 d, 270 d, 365 d	Italy<br>Mixed	Obese, T2DM<br>C: 53.0 ± 6.0<br>T: 51.0 ± 4.0	Unclear<br>ELISA	Protein levels (pg/mL)<br>T: Orlistat+ L-Carnitine (*132*)<br>B: 4.8 ± 2.1<br>A (90 d): 4.3 ± 1.7<br>A (180 d): 3.4 ± 1.1<br>A (270 d): 3.1 ± 0.9<br>A (365 d): 2.8 ± 0.7<br><br>C: Orlistat (*126*)<br>B: 4.4 ± 1.8<br>A (90 d): 4.0 ± 1.5<br>A (180 d): 3.8 ± 1.3<br>A (270 d): 3.7 ± 1.2<br>A (365 d): 3.3 ± 1.0<br><br>T – C (365 d)	 <br>44%<br>50%<br>32%<br>29%<br>25%<br><br><br>41%<br>40%<br>34%<br>32%<br>30%	 <br><br>(NS) (-10%)<br>(*p*<0.05) (-29%)<br>(*p*<0.02) (-35%)<br>(*p*<0.01) (-42%)<br><br><br><br>(NS) (-9%)<br>(NS) (-14%)<br>(NS) (-16%)<br>(*p*<0.05) (-25%)<br><br>-0.9 pg/mL (NS)	Body weight and TNF-α levels decreased in both groups but no significant differences between T and C for the levels of TNF-α. The authors claim that Orlistat + L-Carnitine led to a better improvement in body weight, metabolic profile and inflammatory parameters than Orlistat, but differences were small.
[167]	T: Sibutramine (10 mg/d) + LCD (+ exercise)<br>C: No control group<br>60 d	South Korea<br>Mixed	Obese<br>T: LCD + Sibutramine: 38.5 ± 11.8	Serum<br>ELISA	Protein levels (pg/mL)<br>T: Sibutramine + LCD (+ exercise) (78)<br>B: 2.94 ± 0.9<br>A: 2.48 ± 0.7<br><br>C: NR<br><br>T – C	 <br><br>31%<br>27%	 <br><br>(*p*<0.001)<br>(-16%)<br><br>ND<br><br>ND	Body weight loss upon caloric restriction + Sibutramine (+ exercise) was accompanied by a reduction of TNF-α and improve metabolic risk factors. Since there wasn't a C group, it was not possible to differentiate the LCD effect from the drug effect.
[168]	T1: Sibutramine (10 mg/d)<br>T2: Sibutramine (15 mg/d)<br>RCT (prospective open), 570 d	Turkey<br>Women	Obese<br>T1: 39.6 ± 2.1<br>T2: 40.6 ± 8.1	Plasma<br>ELISA	Protein levels (pg/mL)<br>T1: Sibutramine 10 mg/d (*30*)<br>B: 3.0 ± 1.1<br>A: 2.5 ± 1.09<br><br>T2: Sibutramine 15 mg/d (*30*)<br>B: 3.4 ± 1.5	 <br>37%<br>43.6%<br><br>44%<br>29%	 <br>(*p*<0.04)<br>(-17%,)<br><br>(*p*<0.001)<br>(-30%)	The two doses of Sibutramine reduced body weight and TNF-α levels but no information on significant differences between groups.

					A: 2.4 ± 0.7<br><br>T2 – T1		 <br>-0.5 pg/mL (NR)	It is not addressed if this effect is associated with body weight reduction.
[169]	T: Sibutramine (10 mg/d) + LCD (+ exercise)<br>C: LCD (+ exercise)<br>RCT, 365 d	Italy<br>Mixed	Obese, T2DM<br>C: 53.0 ± 6.0<br>T: 51.0 ± 4.0	Serum<br>NR	Protein levels (pg/mL)<br>T: Sibutramine + LCD + exercise (*110*)<br>B: 4.9 ± 2.2<br>A (90 d): 4.3 ± 1.7<br>A (183 d): 4.2 ± 1.6<br>A (270 d): 3.7 ± 1.3<br>A (365 d): 3.2 ± 1.1<br><br>C: LCD + exercise (*112*)<br>B= 4.5 ± 1.9<br>A (90 d): 4.4 ± 1.8<br>A (183 d): 4.3 ± 1.7<br>A (270 d): 4.1 ± 1.5<br>A (365 d): 3.8 ± 1.4<br><br>T – C (365 d)	 <br><br>45%<br>40%<br>38%<br>35%<br>34%<br><br><br>42%<br>41%<br>40%<br>37%<br>37%	 <br><br><br>(NS) (-12%)<br>(NS) (-14%)<br>($p<0.05$) (-25%)<br>($p<0.01$) (-35%)<br><br><br><br>(NS) (-2%)<br>(NS) (-4%)<br>(NS) (-9%)<br>($p<0.05$) (-16%)<br><br>-1.0 pg/mL (NS)	Body weight reduction more significant in the Sibutramine group.<br>TNF-α levels significantly reduced in both the T and the C groups.<br>TNF-α significant predictor of BMI changes.
[170]	T: Diacerein + LCD + Sibutramine (+ exercise)<br>C: LCD + Sibutramine (+ exercise) (doses not reported)<br>RCT, 84 d	South Korea<br>Mixed	Obese, T2DM, Hypertensive, Dyslipidaemia<br>T: 39.0 ± 1.0<br>C: 37.0 ± 1.0	NR<br>EIA	Protein levels (pg/mL)<br>T: Diacerein + Sibutramine + LCD (*12*)<br>B: 15.7 ± 1.4<br>A: 10.3 ± NR<br><br><br>C: Sibutramine + LCD (7)<br>B: 11.5 ± 2.7<br>A: 5.3 ± NR<br><br>T – C	 <br><br>9%<br>ND<br><br><br><br>23%<br>ND	 <br><br>(NS)<br>(-34%)<br><br><br><br>(NS)<br>(-54%)<br><br>+0.8 pg/mL (NS)	Body weight and TNF-α levels reduction in both groups but no significant differences between them.<br>Diacerein (anti-IL-1 drug), given on top of Sibutramine, did not produce additional weight or metabolic improvement.
[29]	T: Troglitazone (2×200 mg/d)<br>C: PLA<br>RCT (double-blind crossover,), 56 d	The Netherlands<br>Mixed	Obese<br>37.4 ± 1.2	Plasma<br>ELISA	Protein levels (pg/mL)<br>Participants (*126*)<br>T: Troglitazone<br>A: 3.4 ± 0.5	 <br><br>15%	 <br><br>ND	No changes observed for body weight.<br>Lower levels of TNF-α in obese participants treated with troglitazone as compare to PLA

					C: PLA<br>A: 4.5 ± 0.5<br><br>T – C	 11%	 ND<br><br>-1.1 pg/mL (based only on values after intervention)	(↓ -24%, *p*<0.01) but there is no information on baseline values.
[171]	T: Rosiglitazone ± MET (8 mg/d + 1500 ± 500 mg/d) (controlled energy diet)<br>C: MET (2500 ± 500 mg/d) (controlled energy diet)<br>RCT (single-blind placebo-controlled), 90 d, 180 d	Italy<br>Mixed	Overweight/ Obese, T2DM<br>C: 54.0 ± 3.0<br>T: 55.0 ± 4.0	Plasma<br>ELISA	Protein levels (ng/mL)<br>T: Rosiglitazone + MET (*56*)<br>B: 3.2 ± 0.6<br>A (90 d): 2.8 ± 0.5<br>A (180 d): 2.5 ± 0.4<br><br>C: MET (*61*)<br>B= 3.5 ± 0.9<br>A (90 d): 3.3 ± 0.8<br>A (180 d): 3.2 ± 0.7<br><br>T – C (180 d)	 <br>19%<br>18%<br>16%<br><br><br>26%<br>24%<br>22%	 <br><br>(NS) (-13%)<br>(*p*<0.05) (-22%)<br><br><br><br>(NS) (-6%)<br>(NS) (-9%)<br><br>-0.4 pg/mL (NS)	There was no body weight reduction in any group. No significant differences reported between groups for TNF-*α* levels.
[172]	T: HCQ (400 mg/d)<br>C: PLA<br>RCT double-blind, parallel-arm, 93 d	USA<br>Mixed	Overweight/ Obese,<br>IR non-diabetic patients<br>(> 18.0)	NR<br>ELISA	Protein levels (pg/mL)<br>T: HCQ (*13*)<br>B: NR, A: NR<br><br>C: PLA (*15*)<br>B: NR, A: NR<br><br>T – C	 <br><br>ND	 <br><br>No effect<br><br><br><br>ND	There were no significant changes in body weight or any other inflammatory cytokine except for adiponectin.<br>No values reported.
[173]	T: Rimonabant (20 mg/d)<br>C: MET (500 mg 3x/d)<br>RCT (open labelled parallel), 84 d	UK<br>Women	Obese, PCOS<br>NR	Serum<br>Bio-Plex 200 system	Protein levels (pg/mL)<br>T: Rimonabant (*10*)<br>B: 8.7 ± 3.9<br>A: 8.7 ± 3.7<br><br>C: MET (*10*)<br>B: 7.1 ± 2.9<br>A: 7.9 ± 4.3<br><br>T – C	 <br>45%<br>45%<br><br><br>41%<br>54%	 <br>(NS) No change<br><br><br><br>(NS) (+11%)<br><br><br>-0.8 pg/mL (NS)	Significant reduction of body weight with Rimonabant but, no effects on TNF-*α* levels in any group.

[174]	T: Combined Lacidipine (2 and 4 mg) + Candesartan (4, 8, 16 mg)<br>Not a RCT, 56 d	Ukraine<br>Mixed	Overweight/ Obese, Hypertensive<br>T: 54.7 ± 5.8	Serum<br>ELISA	Protein levels (pg/mL)<br>T: combined drugs (*30*)<br>B: 132.6 ± 22.6<br>A: 44.9 ± 5.6<br><br>C: NR<br><br>T – C	 <br>17%<br>13%	 <br>(*p*<0.05)<br>(-66%)<br><br>ND<br><br>ND	It does not indicate results for a control group neither on body weight changes.
[175]	T: MET (dose1:1000, dose2: 2000, dose3: 3000 mg/d)<br>Cross-sectional study	Ghana<br>Mixed	Mostly overweight, T2DM responsive to MET<br>T: 61 to 80 y	Serum<br>ELISA	Protein levels (pg/mL)<br>dose1: 125.0 ± 61.0<br>dose2: 95.0 ± 57.0<br>dose3: 60.0 ± 63.0<br><br>dose3 – dose1	 49%<br>60%<br>>100%	 (*p*<0.0001)<br>(-52%)<br>(dose3 *vs* dose1)<br><br>-65 pg/mL<br>(*p*<0.001)	A significant reduction of TNF-*α* levels with a higher dose.<br>Not an intervention study or clinical trial.<br>No control group.
Protein/peptide drugs								
[176]	T: Etanercept (50 mg ×2 weekly for 90 d, then 50 mg ×1 weekly for 90 d); TNF-*α* inhibitor<br>C: PLA<br>RCT, 180 d	USA<br>Mixed	Obese<br>T: 41.0 ± 2.0<br>C: 47.0 ± 2.0	Serum<br>ELISA	Protein levels (pg/mL)<br>T: Etanercept (*12*)<br>B: 3.4 ± 6.2<br>A (90 d): NR<br>A (180 d): NR<br><br>C: PLA (*22*)<br>B: 0.9 ± 2.3<br>A (90 d): NR<br>A (180 d): NR<br><br>T – C	 <br><br>>100%<br><br><br><br><br>>100%	 <br><br><br>(NS) (+55%)<br><br><br><br>(*p*<0.03) (+51%)<br><br><br>ND	The levels of TNF-*α* were increased in both the T and C groups but no values after treatment reported.<br>No effects on body weight.<br>No significant correlation between BMI and TNF-*α* expression in adipose tissue.
[177]	T: Exenatide (5.0 µg s.c.i. + 0.5 g MET x2/d for 28 d, then 10.0 µg s.c.i. + 0.5 g MET x2/d for 168 d: GLP-1<br>C: No control group included, comparison women *vs* men	China<br>Mixed	Overweight/ Obese, T2DM<br>Females: 49.1 ± 10.4<br>Males: 48.4 ± 9.8	Serum<br>ELISA	Protein levels (pg/mL)<br>Women<br>T: Exenatide (+ MET) (*54*)<br>B: 34.6 ± 85.2<br>A (196 d): 28.0 ± 89.0<br><br>Men<br>T: Exenatide (+ MET) (*51*)<br>B: 33.5 ± 70.0<br>A: 30.3 ± 85.6	 <br><br>>100%<br>>100%<br><br><br>>100%<br>>100%	 <br><br>(*p*=0.001)<br>(-19%)<br><br><br>(*p*=0.039)<br>(-10%)	Reduction of body weight and TNF-*α* levels in both groups but significantly better in women than in men.

					T - C		ND	
[178]	T: Exenatide (5.0 µg s.c.i. + 0.5 g MET x2/d for 30 d, then 10.0 µg s.c.i. + 0.5 g MET x2/d for 90 d: GLP-1<br>C: Acarbose 50-100 mg oral + 0.5 g MET 90 d)<br>RCT (prospective open), 120 d	China<br>Mixed	Obese, T2DM<br>T: 44.4 ± 11.1<br>C: 38.7 ± 10.3	NR<br>NR	Protein levels (pg/mL)<br>T: Exenatide (+ MET) (*15*)<br>B: 25.1 ± 10.7<br>A: 18.1 ± 6.8<br><br>C: Acarbose (+ MET) (*16*)<br>B: 23.2 ± 10.4<br>A: 22.2 ± 8.0<br><br>T – C	43%<br>38%<br><br>45%<br>36%	(*p*<0.001)<br>(-28%)<br><br>(NS)<br>(-4%)<br><br>-6.0 pg/mL (NS)	Exenatide/MET reduced significantly intra-abdominal fat content in comparison with the C group.<br>BMI is reduced in both groups. TNF-α levels significantly reduced with T but difference between the T and C group did not reach significance.
[179]	T: Liraglutide<br>(s. c. i. ×1/d from 0.6 mg to 1.8 mg within 28 d) + MET (orally from 0.5 g ×2/d to 1 g ×2/d within 28 d); GLP-1<br>C: PLA + MET<br>RCT (double-blind cross-over), 84 d	Denmark<br>Mixed	Overweight/ Obese, CAD, T2DM on stable statin therapy<br>62.3 ± 7.6	Plasma<br>ELISA	Protein levels (pg/mL)<br>Patients (*28*)<br>T: Liraglutide<br>B: 6.1 (5.3, 7.0)[d]<br>A: NR<br><br>C: PLA + MET<br>B: NR<br>A: NR<br>-0.2 (-0.6, 0.2)<br><br>T – C	ND<br><br>ND	No effect<br><br>(*p*<0.05)<br>ND<br><br>ND	No significant different results between groups. Lack of data and unclear results but apparent lack of effect of the drug liraglutide on TNF-α levels.<br>No clear information on body weight changes but the authors claim no correlation between TNF-α and body weight changes.

Legend: [a]: Results are presented as the mean ± SD unless otherwise stated; [b]: *N*: group sample size; [c]: CV%: Coefficient of variation ((CV%) = (Standard Deviation/Mean) × 100); [d]: median (IQR); [e]: calculated as ΔT - ΔC; Δ= A – B unless otherwise indicated; A: After treatment; B: Before treatment; C: Control group; CAD: Coronary Artery Disease; d: day; EIA: Enzyme immune assay; GLP-1: Glucagon-like peptide 1; HCQ: Hydroxychloroquine (anti-malarial drug with anti-inflammatory properties); IR: Insulin resistance; LCD: Low Calorie Diet; MET: Metformin; ND: Not determined; NR: Not reported; NS: Not significant; PLA: Placebo; PCOS: Polycistic ovary syndrome; RCT: Randomized controlled trial; SAT: Subcutaneous abdominal adipose tissue; s.c.i.: subcutaneous injection; T: Treatment group; T2DM: Type 2 Diabetes Mellitus; TNF-α: Tumour necrosis factor alpha.
